# ﻿New species of redbait from the Philippines (Teleostei, Emmelichthyidae, *Emmelichthys*)

**DOI:** 10.3897/zookeys.1196.111161

**Published:** 2024-03-22

**Authors:** Matthew G. Girard, Mudjekeewis D. Santos, Katherine E. Bemis

**Affiliations:** 1 Department of Vertebrate Zoology, National Museum of Natural History, Smithsonian Institution, Washington, DC 20560, USA National Museum of Natural History, Smithsonian Institution Washington United States of America; 2 Biodiversity Institute, University of Kansas, Lawrence, KS 66045, USA University of Kansas Lawrence United States of America; 3 Genetic Fingerprinting Laboratory, National Fisheries Research and Development Institute, Quezon City, 1103, Philippines Genetic Fingerprinting Laboratory, National Fisheries Research and Development Institute Quezon City Philippines; 4 National Systematics Laboratory, Office of Science and Technology, NOAA Fisheries, Washington, DC, 20560, USA National Systematics Laboratory, Office of Science and Technology Washington United States of America

**Keywords:** COI, *Erythrocles*, identification key, mitochondrial genome, mitogenome, *Plagiogeneion*, rovers, rubyfishes, systematics, Visayas

## Abstract

We describe a new species of redbait in the genus *Emmelichthys* collected from fish markets on Panay and Cebu islands in the Visayas region of the Philippines. The species is externally similar to *E.struhsakeri* but is diagnosable by two prominent fleshy papillae associated with the cleithrum and fewer pectoral-fin rays (18–19 vs. 19–21) and gill rakers (30–33 vs. 34–41). Additionally, mitochondrial DNA differentiates this taxon from other species of *Emmelichthys*. We generate mitochondrial genomes for two of the three type specimens and several other emmelichthyids to place the new taxon in a phylogenetic context. Analysis of the protein-coding mitochondrial loci calls into question the monophyly of two emmelichthyid genera (*Emmelichthys* and *Erythrocles*) and highlights the need for subsequent analyses targeting the intrarelationships of the Emmelichthyidae.

## Abstract (tl)

Dito pinakita namin ang isang kakaibang isda na may Tagalog name na Rebentador pula at English name na Redbait na kabilang sa genus *Emmelichthys* na nakuha sa mga pamilihan ng isda sa isla ng Panay at Cebu sa Visayas, Philippines. Ang isdang ito ay may panglabas na anyo kamukha ng *E.struhsakeri* pero naiba ito dahil meron itong dalawa (2) prominenteng fleshy papillae na parte ng cleithrum, may mas konting pectoral-fin rays na may bilang na 18–19 at gill rakers na may bilang na 30–33. Iniiba ng mitochondrial DNA ang taxon na ito mula sa iba pang mga species ng *Emmelichthys*. Binuo, sinuri at kinumpara namin ang mitochondrial genomes ng dalawang type specimens ng kakaibang isda at iba pang isda na kabilang sa emmelichthyids para malaman kung bago nga ba ito. Lumabas sa pagsusuri, gamit ang lahat ng protein-coding mitochondrial loci, na bago nga ang kakaibang isda. Pero napag-alaman din na mukhang isang grupo lang at malapit na mag kamag-anak ang 2 genus (*Emmelichthys* and *Erythrocles*) na kasama sa Family Emmelichthyidae kung kaya’t kailangan pa ang ibayong pagsusri sa pagkakakilanlan ng nasabing 2 genus.

## ﻿Introduction

The Emmelichthyidae is a small family of fishes found in all temperate and tropical oceans between depths of 100 and 400 m. Commonly known as rovers, redbaits, and rubyfishes, emmelichthyids are often bright red in color and can be distinguished from other fishes by their fusiform bodies, highly protrusible mouths, toothless or nearly toothless jaws, and large rostral cartilage (Heemstra and Randall 1977; Johnson 1980). Little is known about the life history of emmelichthyids, with a recent study documenting larvae and juveniles of some species feeding within and around pelagic tunicates (Pastana et al. 2022). The family currently includes 17 species in three genera: *Emmelichthys*, *Erythrocles* and *Plagiogeneion* (Fricke et al. 2023; Girard 2024). Among emmelichthyids, the genus *Emmelichthys* is diagnosed by a highly fusiform body and separation of the spinous and soft dorsal fins by a distinct gap that contains one or more isolated dorsal-fin spines (Heemstra and Randall 1977). Six species are included in the genus: *E.cyanescens* (Guichenot, 1848) [recognized as a species by Fricke et al. (2014) but see study by Oyarzún and Arriaza 1993], *E.elongatus* Kotlyar, 1982, *E.karnellai* Heemstra & Randall, 1977, *E.nitidus* Richardson, 1845, *E.ruber* (Trunov, 1976) and *E.struhsakeri* Heemstra & Randall, 1977. A seventh species was described by Fricke et al. (2014) but this taxon has been found to be a species of *Dipterygonotus* in the Lutjanidae [“*Emmelichthys*” *marisrubri* = *Dipterygonotusmarisrubri* (Fricke, Golani & Appelbaum-Golani, 2014); see Girard 2024]. Although a phylogeny of *Emmelichthys* and the Emmelichthyidae has yet to be generated, Heemstra and Randall (1977) noted morphological similarities and suggested relationships among species. For example, they considered *E.cyanescens* and *E.nitidus* to be closely related based on the presence of prominent protuberances on the anterior margin of the cleithrum (hereafter referred to as cleithral papillae).

In 2011, a collaboration among researchers at the National Museum of Natural History, Smithsonian Institution (NMNH), the Bureau of Fisheries and Aquatic Resources−National Fisheries Research and Development Institute, Department of Agriculture, Philippines (BFAR−NFRDI), and United States Food and Drug Administration (FDA) was established to document the diversity of fishes in Philippine markets. The goal of this collaboration was to develop a voucher-based genetic reference library to advance consumer safety and biodiversity research (Bemis et al. 2023). The project has yielded descriptions of several new species (e.g., Williams and Carpenter 2015; Carpenter et al. 2017; Matsunuma et al. 2018) and discovered additional taxa that have yet to be described (see Bemis et al. 2023). Two emmelichthyid specimens were collected from a fish market on Cebu Island in 2013 that are externally similar to *E.struhsakeri*, but they have two prominent fleshy papillae associated with the cleithrum, fewer pectoral-fin rays, and fewer gill rakers. While reviewing additional specimens, we identified a third Philippine specimen purchased at a fish market on Panay Island in 2016 that has the same phenotype as the two specimens from 2013. Examination of both genotypic and phenotypic characters of these papillae-bearing specimens indicates they represent an undescribed species. We describe this species and generate a phylogeny based on mitochondrial loci to place the taxon in an evolutionary context.

## ﻿Materials and methods

### ﻿Specimen examination

Methods for counts and measurements follow Heemstra and Randall (1977). Standard length is abbreviated as SL; total length is abbreviated as TL. Museum abbreviations follow Sabaj (2020) except for NMNH, which refers to non-Fishes Division equipment and personnel at the National Museum of Natural History, Smithsonian Institution. All specimens examined in this study, along with their lengths and museum catalog numbers, are listed in Table 1.

**Table 1.** Specimens examined in this study.

Species	Museum voucher	Count	Collection latitude, longitude	SL (mm)	MorphoSource
***Emmelichthyspapillatus* sp. nov. holotype**	PNM 15806	1	11.000, 123.000	130	554144
***Emmelichthyspapillatus* sp. nov. paratype**	USNM 424606	1	10.292, 123.892	122	553712
***Emmelichthyspapillatus* sp. nov. paratype**	KAUM-I. 193858	1	10.292, 123.892	119	553717
*Emmelichthyskarnellai*	KAUM-I. 146310	1		212	
*Emmelichthyskarnellai*	KAUM-I. 149380	1	28.467, 129.467	208	
*Emmelichthyskarnellai* paratype	USNM 214689	1	21.260, -157.207	101	553688
*Emmelichthysnitidus*	CSIRO H4244-01	1	-38.188, 149.277	274	553698
*Emmelichthysnitidus*	NSMT P.125978	16	-32.355, 130.035	116–123	553651
*Emmelichthysstruhsakeri* holotype	USNM 214690	1	20.722, -156.830	150	553667
*Emmelichthysstruhsakeri* paratype	USNM 214691	10	20.722, -156.830	136–159	
*Emmelichthysstruhsakeri* paratype	AMS I.17244-001	1	-34.330, 151.000	170	
*Emmelichthysstruhsakeri*	KAUM-I. 149520	1	28.467, 129.467	216	
*Erythroclesmicroceps*	NSMT P.102428	10		68–80	
*Erythroclesschlegelii*	NSMT P.105302	1		119	
*Erythroclesschlegelii*	USNM 403355	1	9.199, 123.267	230	
*Erythroclesscintillans*	OCF-P. 3558	1			
*Erythroclesscintillans* holotype	USNM 51051	1		282	
*Plagiogeneionmacrolepis*	CSIRO H8671-01	1	-41.177, 144.192	215	
*Plagiogeneionrubiginosum*	NZ P.045174	1	-44.178, -176.955	194	

### ﻿Specimen imaging

We used microcomputed tomography (µCT) to examine internal osteology. Specimens were scanned using a GE Phoenix v|tome| x M 240/180 kV Dual Tube μCT at NMNH. Scan settings were 120–130 kV, 150 µA, 250 ms exposure time, and 34–60 µm voxel size. Resulting scans are available through MorphoSource project ID 000553669 and media identifiers for individual specimens can be found in Table 1. Scans of additional species generated in a previous study (project ID 000553611; Girard 2024) were also downloaded from MorphoSource for examination. All scan data were visualized and segmented using the protocol in Girard et al. (2022a). All other specimen imaging was performed using equipment and protocols listed in Girard et al. (2020) and Bemis et al. (2023).

### ﻿Extraction, sequencing, assembly, and annotation of genetic data

We extracted genomic DNA from 13 samples of the Emmelichthyidae. These include the new species described in this study, three species of *Emmelichthys* (*E.karnellai*, *E.nitidus*, and *E.struhsakeri*), three species of *Erythrocles* [*E.microceps* Miyahara & Okamura, 1998, *E.schlegelii* (Richardson, 1846), and *E.scintillans* (Jordan & Thompson, 1912)], and two species of *Plagiogeneion* [*P.macrolepis* McCulloch, 1914 and *P.rubiginosum* (Hutton, 1875)]. Protocols for DNA extraction follow the methods described in Weigt et al. (2012). For 12 samples, we sequenced whole mitochondrial genomes (hereafter, mitogenomes) using the library preparation and sequencing protocol described in Hoban et al. (2022). Demultiplexed sequence data received in compressed FASTQ format were cleaned of adapter contamination and low-quality bases using the parallel wrapper illumiprocessor version 2.10 (Faircloth 2013) around trimmomatic version 0.39 (Bolger et al. 2014). Cleaned reads were submitted to GenBank and assigned SRA accession numbers SRR27284234–SRR27284245 under BioProject PRJNA1052721 (see Table 2). We assembled mitogenomes using the ‘map to reference’ function in Geneious version 11.1.5 (Kearse et al. 2012) with the settings described in Girard et al. (2022b) and a reference mitogenome downloaded from GenBank (*E.struhsakeri*, GenBank NC_004407; Miya et al. 2003). Assembled mitogenomes were annotated using MitoAnnotator (Iwasaki et al. 2013; Sato et al. 2018; Zhu et al. 2023). Annotated mitogenomes were submitted to GenBank and assigned accession numbers OR974326–OR974337 (see Table 2). For one paratype (KAUM-I. 193858 [ex. USNM 424607]) only the cytochrome oxidase I barcode sequence was generated following the methods described in Weigt et al. (2012) and using the primers from Baldwin et al. (2009). The sequence contig was built, edited, and assembled using Geneious and deposited in GenBank (OR961526; see Table 2).

**Table 2.** Genetic voucher and GenBank information for samples examined in this study.

Species	Museum voucher	GenBank SRA	GenBank mitogenome accession number	GenBank COI accession number
***Emmelichthyspapillatus* sp. nov. holotype**	PNM 15806	SRR27284241	OR974328	See mitogenome
***Emmelichthyspapillatus* sp. nov. paratype**	USNM 424606	SRR27284240	OR974329	See mitogenome
***Emmelichthyspapillatus* sp. nov. paratype**	KAUM-I. 193858			OR961526
*Emmelichthyskarnellai*	KAUM-I. 146310	SRR27284245	OR974326	See mitogenome
*Emmelichthyskarnellai*	KAUM-I. 149380	SRR27284244	OR974327	See mitogenome
*Emmelichthysnitidus*	CSIRO H4244-01	SRR27284239	OR974330	See mitogenome
*Emmelichthysstruhsakeri*	KAUM-I. 149520	SRR27284238	OR974331	See mitogenome
*Emmelichthysstruhsakeri*			NC_004407	See mitogenome
*Erythroclesmicroceps*	NSMT P.102428	SRR27284237	OR974332	See mitogenome
*Erythroclesschlegelii*	NSMT P.105302	SRR27284236	OR974333	See mitogenome
*Erythroclesschlegelii*	USNM 403355	SRR27284235	OR974334	See mitogenome
*Erythroclesscintillans*	OCF-P. 3558	SRR27284234	OR974335	See mitogenome
*Plagiogeneionmacrolepis*	CSIRO H8671-01	SRR27284243	OR974336	See mitogenome
*Plagiogeneionrubiginosum*	NZ P.045174	SRR27284242	OR974337	See mitogenome

### ﻿Phylogenetic analysis

To generate a hypothesis of relationships for the taxa sampled in our study, we collated orthologous loci from the 13 protein-coding regions of the mitogenome into individual FASTA files and aligned them with MAFFT version 7 (Katoh and Standley 2013). Lengths of alignments were as follows: ATPase6 683 base pairs (bps); ATPase8 168 bps; COI 1551 bps; COII 691 bps; COIII 785 bps; CytB 1141 bps; ND1 975 bps; ND2 1046 bps; ND3 349 bps; ND4 1381 bps; ND4L 297 bps; ND5 1839 bps; ND6 522 bps. Aligned matrices were concatenated for partitioning and phylogenetic inference. IQ-Tree version 2.2.0 (i.e., MFP + MERGE; Chernomor et al. 2016; Kalyaanamoorthy et al. 2017; Minh et al. 2020) recovered an optimal partitioning scheme of six groups based on 39 partitions designated for the three codon positions in each of the loci. Ten tree searches were performed in IQ-Tree using the optimal partitioning scheme and concatenated alignment. Support for the resulting topology was assessed by generating 500 standard bootstrap replicates (-bo). Analyses were rooted on *Plagiogeneionrubiginosum*.

## ﻿Results

### ﻿Species description

#### ﻿ Emmelichthys papillatus sp. nov.

Taxon classificationAnimaliaPerciformesEmmelichthyidae

BABCF9F1-326F-5D68-8A43-5C3D2FF327EF

https://zoobank.org/804D459E-72C9-458A-A347-6CD8D88B2E30

##### Etymology.

Named for the diagnostic fleshy cleithral papillae.

##### English name.

Papillated redbait.

##### Tagalog name.

Rebentador pula.

##### Types.

**Figure 1.**
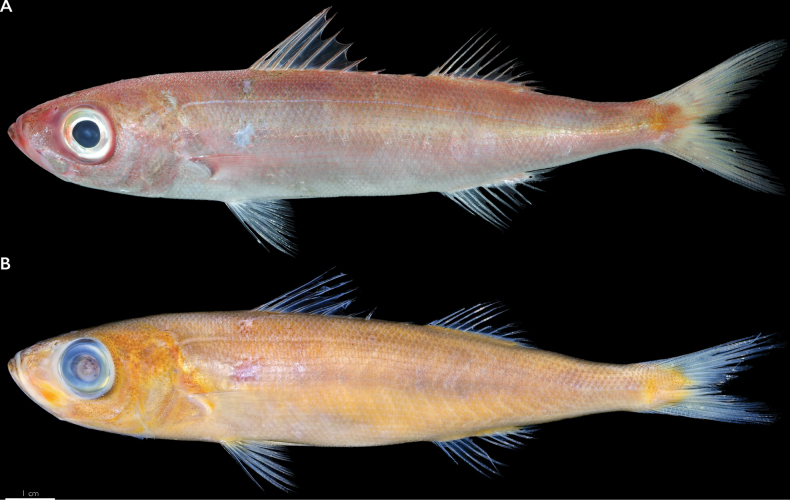
Holotype of *Emmelichthyspapillatus* sp. nov. (PNM 15806 [ex. KAUM-I. 91845]) from the Philippines **A** before preservation. Photograph by the Kagoshima University Museum **B** preserved specimen.

**Figure 2.**
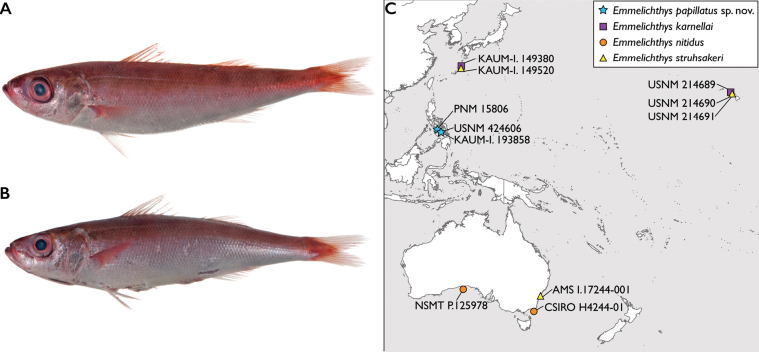
Paratypes of *Emmelichthyspapillatus* sp. nov. and collection localities for specimens examined in this study **A** KAUM-I. 193858 (ex. USNM 424607) before preservation **B** USNM 424606 before preservation. Photographs by J. T. Williams **C** distribution of Pacific *Emmelichthys* spp. type materials examined.

***Holotype*.** PNM 15806 (ex. KAUM-I. 91845); 154 mm TL; 130 mm SL; purchased 12 September 2016 from Oton Fish Market; likely captured off Iloilo, Panay Island, Philippines, 11°N, 123°E (Fig. 1, Tables 1–4). Collected by Y. Fukui and M. Matsunuma (Motomura et al. [2017: 128] identified as *E.struhsakeri*). ***Paratypes*.** USNM 424606; 138 mm TL; 122 mm SL; purchased 1 June 2013 from Pasil Market, Cebu Island, Philippines, 10°17'30.1"N, 123°53'31.2"E (Fig. 2, Tables 1–4). Collected by J. T. Williams, K. E. Carpenter, A. Lizano, and A. Macaspac. KAUM-I. 193858 (ex. USNM 424607); 132 mm TL; 119 mm SL; same collection information as USNM 424606.

##### Diagnosis.

**Figure 3.**
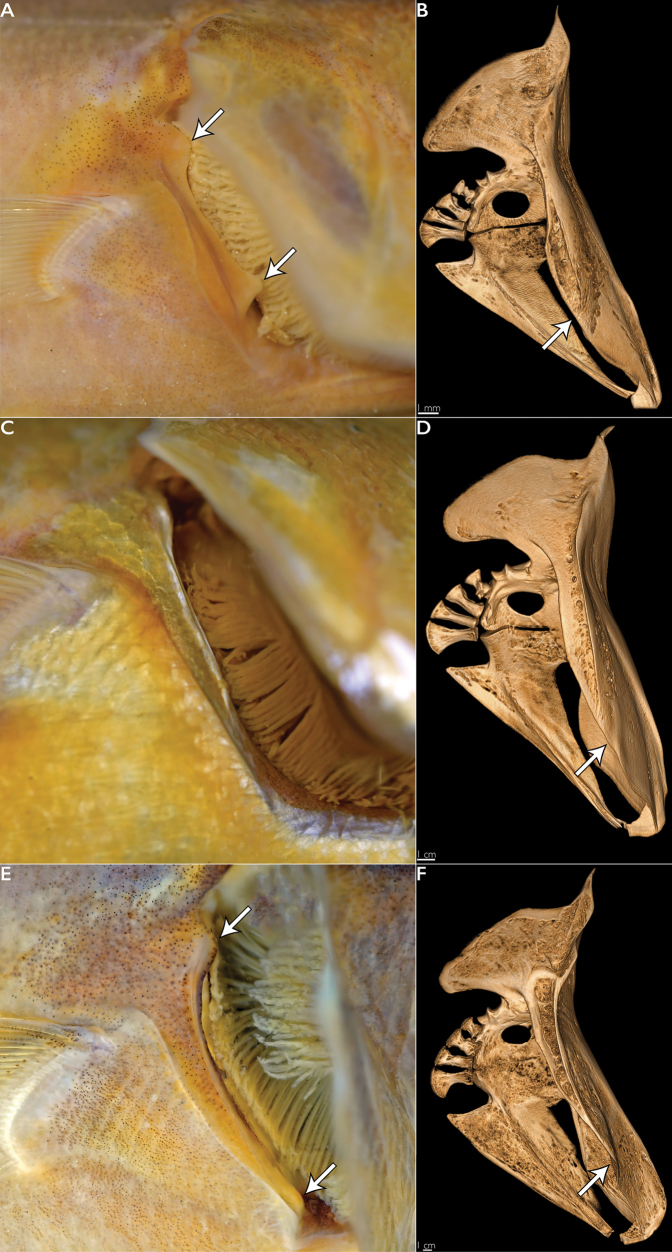
Pectoral girdle in species of *Emmelichthys***A** fleshy cleithral papillae (arrows) in *E.papillatus* sp. nov. (PNM 15806 [ex. KAUM-I. 91845] holotype) **B**µCT scan of pectoral girdle in *E.papillatus* sp. nov. (PNM 15806 [ex. KAUM-I. 91845] holotype). Arrow indicates absence of anterior expansion of cleithrum **C** absence of cleithral papillae in *E.struhsakeri* (AMS I.17244-001) **D**µCT scan of pectoral girdle in *E.struhsakeri* (USNM 214690 holotype). Arrow indicates absence of anterior expansion of cleithrum **E** bony cleithral papillae (arrows) in *E.nitidus* (NSMT P.125978) **F**µCT scan of pectoral girdle in *E.nitidus* (CSIRO H 4244-01). Arrow indicates prominent anterior expansion of cleithrum that supports ventral cleithral papilla.

*Emmelichthyspapillatus* is distinguished from congeners in the Pacific Ocean by the presence of two fleshy papillae on the cleithrum (absent in *E.elongatus*, *E.karnellai*, *E.struhsakeri*; see Fig. 3) and fewer number of gill rakers (30–33 vs. 34+ in other species). It can be further differentiated from *E.cyanescens* and *E.nitidus*, which have bony cleithral papillae, by fewer pectoral-fin rays (18–19 vs. 22 in *E.cyanescens*, 20–23 in *E.nitidus*) and fewer lateral-line scales (69–74 vs. 100–105 in *E.cyanescens*, 87–93 in *E.nitidus*). It can also be differentiated from *Erythroclesschlegelii*, which also has fleshy cleithral papillae, by II isolated dorsal-fin spines between the spinous and soft dorsal fin.

##### Description

**(See Tables 3, 4 for counts and measurements).** Dorsal fin with anterior VIII spines connected by membrane; penultimate II spines not connected to adjacent spines via membrane but with short membrane behind each spine; membrane of last dorsal-fin spine connected to first soft dorsal-fin ray. Upper 2 pectoral-fin rays unbranched. Body and head, except for a narrow median region dorsal to upper lip, covered with ctenoid scales; 5–7 scales from middle of spinous dorsal fin to lateral line; 7–8 scales from dorsal-fin origin and 14–16 from anal-fin origin to lateral line; 26–28 circumpeduncular scales. Soft dorsal and anal fins with scaly sheath at base, broadening near last few rays; no scales on dorsal or anal fins beyond basal sheath; pectoral fins scaled proximally; caudal fin with small scales on basal fleshy region and proximally on rays. Nostrils small, subequal, close-set. Maxilla reaching vertical at front edge of pupil. Opercle with 2–3 flat spines. No teeth on vomer, palatines, or jaws. Shallow groove on rear margin of gill cavity at upper end of cleithrum; cleithrum with two pronounced fleshy papillae that lack underlying osteological support (Fig. 3A–B; compare with *E.struhsakeri* [Fig. 3C–D] and *E.nitidus* [Fig. 3E–F]). Pectoral fins reaching slightly posterior to vertical at tips of pelvic fins. Anal fin origin slightly posterior to vertical at first soft dorsal-fin ray. Anus well in advance of anal fin origin.

**Table 3.** Counts and measurements of type specimens for *Emmelichthyspapillatus* sp. nov. Dashes indicate data not collected because of specimen damage.

Characters	Holotype	Paratype	Paratype
	PNM 15806	USNM 424606	KAUM-I. 193858
Total length in mm	154	138	132
Standard length (SL) in mm	130	122	119
Dorsal-fin spines	XI	–	XI
Dorsal-fin spines connected by membrane	VIII	–	VIII
Isolated posterior dorsal-fin spines	II	II	II
Dorsal-fin rays	11	11	11
Pectoral-fin rays	18	19	19
Anal-fin rays	10	10	10
Gill rakers (Upper + Lower)	8+22	8+25	8+25
Lateral-line scales	74	74	69
Fleshy cleithral papillae	Present	Present	Present
Body depth in %SL	19.8	–	–
Body width in %SL	12.5	11.6	–
Head length in %SL	27.9	26.0	25.3
Orbit diameter in %SL	7.7	7.0	6.7
Interorbital width in %SL	7.4	6.3	7.1
Predorsal distance in %SL	36.2	34.4	35.3
Distance from snout to anus in %SL	60.9	–	–
Spinous dorsal-fin base in %SL	27.8	27.5	27.4
Pectoral-fin length in %SL	17.7	15.7	16.3
Pelvic-fin length in %SL	14.6	13.1	12.6
Caudal-peduncle depth in %SL	7.2	7.8	7.3
Caudal-peduncle width in %SL	3.5	3.0	4.1
Longest dorsal-fin spine in %SL	13.1	12.6	12.4
Penultimate dorsal-fin spine in %SL	2.1	2.8	1.9
Last dorsal-fin spine in %SL	–	3.2	3.2
First anal-fin spine in %SL	1.3	–	1.4
Third anal-fin spine in %SL	4.5	–	5.2
Pelvic base to anus in %SL	28.1	–	–

**Table 4.** Counts and measurements among species of *Emmelichthys*. Values for species not described in this study from Heemstra and Randall (1977), Kotlyar (1982) and Fricke et al. (2014). Dashes indicate data not available.

Characters	*E.papillatus* sp. nov.	*E.cyanescens*	*E.elongatus*	*E.karnellai*	*E.nitidus*	*E.ruber*	*E.struhsakeri*
Dorsal-fin spines	XI	XIII–XIV	XII	XII–XIII	XIII–XIV	XII–XIII	XI–XII
Dorsal-fin spines connected by membrane	VIII	XI–X	VIII	VIII–IX	IX–X	VII–IX	VIII–X
Isolated posterior dorsal-fin spines	II	II–III	III	IV–V	II–III	III–V	I–III
Length of posterior dorsal-fin spines	Protruding	Protruding	Protruding	Embedded	Protruding	Embedded	Protruding
Dorsal-fin rays	11	9–10	9–10	10–11	9–11	9–11	10–12
Pectoral-fin rays	18–19	22	18–20	21–23	20–23	19–20	19–21
Anal-fin rays	10	10–11	9–10	9–10	9–10	9–10	9–10
Gill rakers	30–33	39–42	34–38	37–43	37–43	33–38	34–41
Lateral-line scales	69–74	100–105	61–68	76–85	87–98	71–74	68–76
Cleithral papillae	Present - Fleshy	Present - Bony	Absent	Absent	Present - Bony	Absent	Absent
Body depth in %SL	19.8	18.0–22.0	15.0–19.0	19.0–22.0	19.0–24.0	19.0–28.0	20.0–25.0
Body width in %SL	11.6–12.5	–	11.0–13.0	14.0–17.0	11.0–17.0	11.0–16.0	13.0–16.0
Head length in %SL	25.3–27.9	25.0–27.0	26.0–27.0	25.0–27.0	25.0–30.0	25.0–32.0	26.0–30.0
Orbit diameter in %SL	6.7–7.7	7.1–8.7	6.5–9.6	8.8–9.6	7.0–11.0	8.6–12.9	9.0–11.1
Interorbital width in %SL	6.3–7.4	5.9–6.2	5.4–6.6	7.0–7.7	6.0–7.7	5.8–7.1	6.3–7.8
Predorsal distance in %SL	34.4–36.2	35.0–37.0	–	37.0–39.0	35.0–39.0	35.0–43.0	35.0–40.0
Distance from snout to anus in %SL	60.9	64.0–67.0	–	57.0–66.0	64.0–72.0	57.0–62.0	58.0–63.0
Spinous dorsal-fin base in %SL	27.4–27.8	30.0–31.0	28.0–36.0	32.0–34.0	30.0–36.0	25.0–31.0	26.0–30.0
Pectoral-fin length in %SL	15.7–17.7	18.0–20.0	16.0–20.0	17.0–19.0	19.0–24.0	16.0–20.0	18.0–21.0
Pelvic-fin length in %SL	12.6–14.6	13.0–14.0	10.0–14.0	11.0–15.0	13.0–17.0	12.0–20.0	14.0–16.0
Caudal-peduncle depth in %SL	7.2–7.8	6.0–7.1	5.8–7.5	5.7–7.7	6.5–8.5	6.3–11.6	6.4–8.3
Caudal-peduncle width in %SL	3.0–4.1	–	5.2–7.2	4.2–4.9	2.8–5.7	–	3.0–5.5
Longest dorsal-fin spine in %SL	12.4–13.1	12.0	–	12.0–16.0	12.0–15.0	12.0–15.0	13.0–16.0
Penultimate dorsal-fin spine in %SL	1.9–2.8	2.9	–	2.6–3.7	2.5–3.8	0.6–1.3	2.1–3.8
Last dorsal-fin spine in %SL	3.2	2.5	–	3.3–4.1	2.1–3.7	3.1–3.6	3.1–5.5
First anal-fin spine in %SL	1.3–1.4	1.5–1.9	1.1–2.4	1.0–1.9	1.0–2.9	1.2–3.8	1.4–2.8
Third anal-fin spine in %SL	4.5–5.2	4.2–5.3	2.7–6.0	4.1–6.4	3.1–6.7	4.8–7.1	4.7–7.3
Pelvic base to anus in %SL	28.1	–	25.0–30.0	8.0–11.0	15.0–27.0	–	9.0–14.0

Color of market specimens dusky rose dorsally, becoming silver-pink ventrally (Figs 1–2). Indistinct wide lateral bar of yellowish pink below lateral-line canal. Indistinct dark mottling above the lateral-line canal. Centers of flank scales darker pink. Dorsal fin pinkish white; pelvic, anal, and caudal fins whitish, with rays pinker than membrane; pectoral fins pink, grading to white distally; lips red. In alcohol, uniformly tan, no distinct coloration remains (Fig. 1).

##### Distribution.

All three specimens of *Emmelichthyspapillatus* were collected from markets of the Visayas region of the Philippines (Fig. 2C). It is unknown if this species occurs beyond Philippine waters.

### ﻿Mitochondrial data

**Figure 4.**
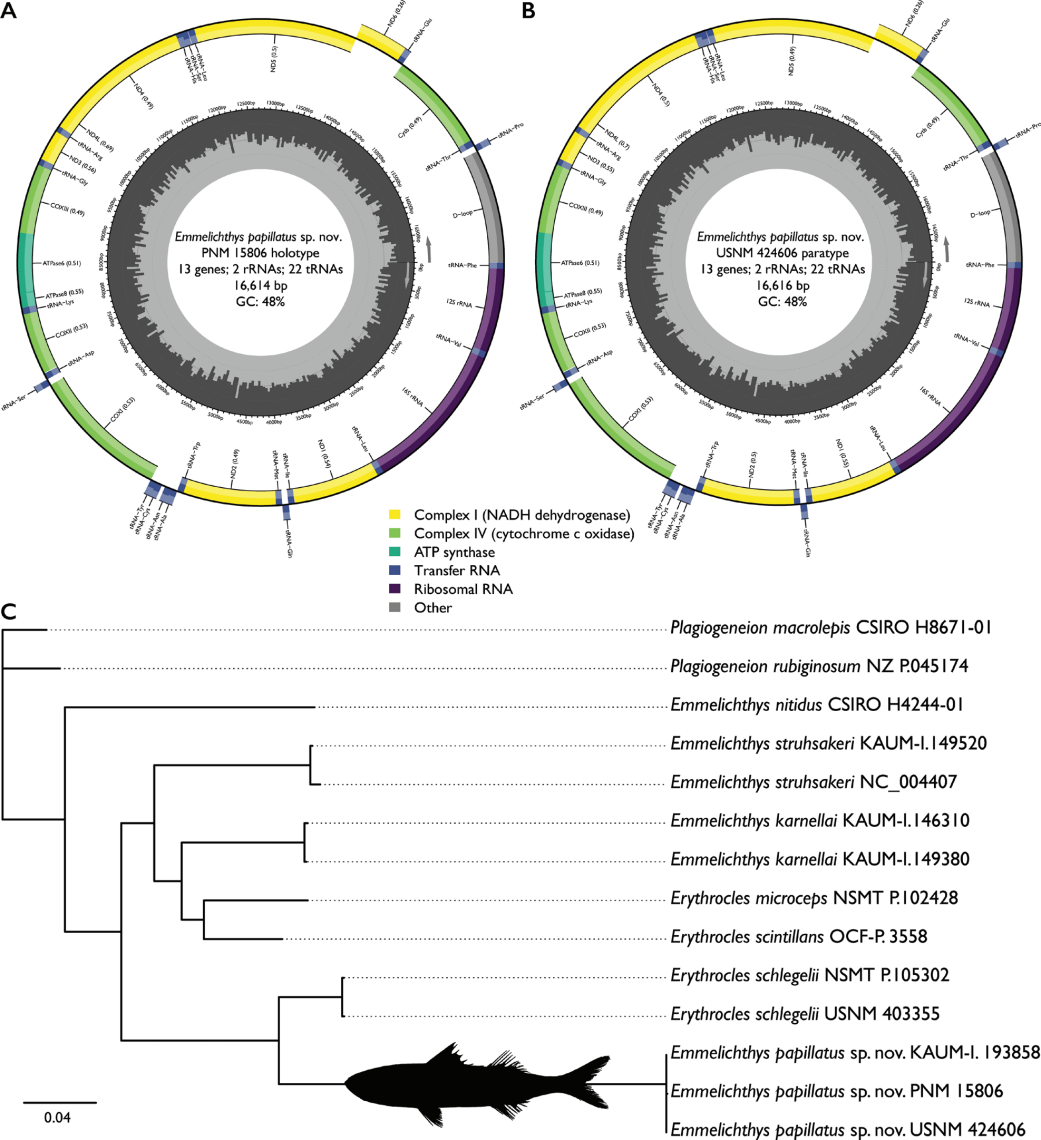
Mitogenome structure and placement of *E.papillatus* sp. nov. among species of *Emmelichthys***A** mitogenome structure of *E.papillatus* sp. nov. (PNM 15806 [ex. KAUM-I. 91845] holotype) **B** mitogenome structure of *E.papillatus* sp. nov. (USNM 424606 paratype) **C** phylogeny of emmelichthyids based on 13 protein-coding mitochondrial loci. Bootstrap values not listed, see text.

Mitogenomes of two type specimens are circular and 16,614–16,616 bps in length (99.9% similar; 9 bps different total). Both encoded 37 mitochondrial loci (13 protein coding, 22 tRNAs, and 2 rRNAs) and one non-coding control region (D-loop). Of these, 26 loci are on the majority strand and the remaining nine are on the minority strand. The locus order matches that of previously sequenced species of *Emmelichthys* (Fig. 4; Miya et al. 2003). Sequences of *E.papillatus* are 82.5–87.5% similar (2046–2964 bps different total) to all other emmelichthyids sampled in this study. The COI barcode from the three types is 99.85–100% similar (1 bp different total), with sequences of *E.papillatus* 88.5–91.3% similar (130–184 bps different total) from all other emmelichthyids sampled in this study.

### ﻿Results of phylogenetic analysis

All ten tree searches resulted in a single optimal topology with slightly different branch lengths. The best-scoring topology (Ln *L* = –37445.526) is shown in Fig. 4. High levels of support were recovered, with all but one node having a value ≥97% (Fig. 4). We recovered all three specimens of *E.papillatus* in an independent lineage from other species of *Emmelichthys* sampled (i.e., *E.karnellai*, *E.nitidus* and *E.struhsakeri*). *Emmelichthys* is recovered as a non-monophyletic group, with species of *Erythrocles* nested among the species of *Emmelichthys*. *Emmelichthysnitidus* is the earliest-diverging species, with the remaining taxa sampled recovered in two clades. In one clade, *Emmelichthysstruhsakeri* is the earliest-diverging species, with *Emmelichthyskarnellai* sister to a clade of *Erythroclesmicroceps* and *Erythroclesscintillans*. In the other clade, all samples of *E.papillatus* are recovered sister to all samples of *Erythroclesschlegelii* (Fig. 4).

## ﻿Discussion

### ﻿Fishery implications

We did not identify additional specimens of *Emmelichthyspapillatus* in collections beyond the three type specimens described in this study. This may be due, in part, to the rarity of emmelichthyids housed in museums, a lack of species-specific identification of freshly caught specimens, and/or the challenges of species-specific identifications for emmelichthyids broadly. In the Philippines, species of *Emmelichthys* are caught by bagnet, Danish seine, fish corrals, hook and line, otoshi ami, purse seine, ringnet, stationary liftnet, and trawl, but are not typically identified to species (Calvelo et al. 1991). Locally known as rebentador, sikwan and tuliloy, species of *Emmelichthys* are sold in markets, especially in Caraga, Cebu and Panay. It is unknown what percentage of *Emmelichthys* spp. catch in the Philippines is *E.papillatus*.

### ﻿Non-monophyly of *Emmelichthys* and *Erythrocles*

When compared with species of *Plagiogeneion*, species of *Emmelichthys* and *Erythrocles* have divided spinous and soft dorsal fins and more fusiform bodies (see Heemstra and Randall 1977). Along with the morphology of the dorsal fin, Heemstra and Randall (1977) further separated the genera of emmelichthyids by differences in head length and body depth; however, we lack a phylogenetic assessment targeting emmelichthyid intrarelationships. Rabosky et al. (2018) included one species of *Emmelichthys* (*E.nitidus*), two species of *Erythrocles* (*E.monodi* Poll & Cadenat, 1954 and *E.schlegelii*) and two species of *Plagiogeneion* (*P.macrolepis* and *P.rubiginosum*) in their study on the broad relationships among ray-finned fishes, recovering *Erythrocles* as non-monophyletic based on five overlapping loci (see their suppl. materials). Similarly, we recovered a non-monophyletic *Erythrocles* in our study as well as a non-monophyletic *Emmelichthys*. As the intrarelationships among emmelichthyids are beyond the scope of this study, we do not modify the classification of the family based on our dataset. Morphological convergence has caused confusion about the taxonomy and classification of the Emmelichthyidae for nearly 80 years (see Schultz 1945; Heemstra and Randall 1977; Johnson 1980; Girard 2024) and the dorsal-fin morphology and differences in head length and body depth that diagnose *Emmelichthys*, *Erythrocles* and *Plagiogeneion* may have repeatedly evolved within the family. Subsequent investigations into the intrarelationships of Emmelichthyidae are needed to understand the evolution of these and other morphological characters of rovers, redbaits and rubyfishes.

### ﻿Key to the species of *Emmelichthys* (modified from Heemstra and Randall [1977] and Fricke et al. [2014])

**Table:** 

1	Posterior dorsal-fin spines embedded within dorsal profile of body	**2**
–	Posterior dorsal-fin spines protruding above dorsal profile of body	**3**
2	Lateral-line scales 71–74; pectoral-fin rays 19–20; total gill rakers 33–38	***E.ruber* (Bermuda, Jamaica and St. Helena)**
–	Lateral-line scales 76–85; pectoral-fin rays 21–23; total gill rakers 37–43	***E.karnellai* (Hawaiian Islands and Easter Island)**
3	Lateral-line scales 61–76	**4**
–	Lateral-line scales 87–105	**6**
4	Lateral-line scales 61–68; body depth 15.0–19.0% SL	***E.elongatus* (Nazca Ridge and Southeastern Pacific Ocean)**
–	Lateral-line scales 68–76; body depth 19.8–25.0% SL	**5**
5	Pectoral-fin rays 18–19; gill rakers 30–33; fleshy cleithral papillae present	***E.papillatus* sp. nov. (Philippines)**
–	Pectoral-fin rays 19–21; gill rakers 34–41; cleithral papillae absent	***E.struhsakeri* (Australia, Hawaiian Islands and Japan)**
6	Lateral-line scales 87–98	***E.nitidus* (Australia, New Zealand, St. Paul and Amsterdam Islands and South Africa)**
–	Lateral-line scales 100–105	***E.cyanescens* (Chile and Juan Fernandez Islands)**

## Supplementary Material

XML Treatment for Emmelichthys papillatus

## ﻿Additional information

### Author ORCIDs

Matthew G. Girard https://orcid.org/0000-0003-3580-6808

Mudjekeewis D. Santos https://orcid.org/0000-0002-4770-1221

Katherine E. Bemis https://orcid.org/0000-0002-7471-9283

### Data availability

All of the data that support the findings of this study are available in the main text, on GenBank, and/or MorphoSource.
